# Genetic analysis of X-chromosomal short tandem repeat (X-STR) frequencies in Arab Iraqi male population

**DOI:** 10.1186/s43141-022-00403-7

**Published:** 2022-08-06

**Authors:** Salwa J. Al-Awadi, Hayder A. Khaleefah, Shaimaa Y. Abdulfattah

**Affiliations:** 1 College of Biotechnology, Al-Nahrain University, Baghdad, Iraq; 2 Biotechnology Research Center/ Al-Nahrain University, Baghdad, Iraq

**Keywords:** X-STR, Marker, Haplotype, Polymorphism, X-chromosome

## Abstract

**Background:**

The X-chromosome short tandem repeat (STR) polymorphisms are a particular tool in the fields of human population genetics and personal identification. It was necessary in investigating complex kinship or deficiency cases in conditions where information on mitochondrial DNA (mtDNA) or Y chromosome polymorphisms have been used to explore their direct paternal line. This study aimed to investigate the allele frequency of (12X-STR) of 200 unrelated males from different region of Baghdad City to serve as a reference data base for individual identification in Iraqi population.

**Results:**

Twelve X-STR loci (DXS7424, HPRTB, DXS8377, GATA31E08, DXS7423, DXS8378, DXS9895, DXS10074, DXS6809, DXS7133, DXS101, DXS6807) were successfully amplified by multiplex PCR and divided into four groups. According to measures of allele frequency, the higher alleles frequency were 16, 11, 46, 11, 14, 10, 15, 15.2, 35, 11, 25, and 11 while the lowest alleles frequency were 11, 9, 52,53, 7, 17, 14, 13, 12.2,17, 36, 15, 16, 22, 29, and 17 that observed at the 12 loci respectively. Forensic efficiency parameter for DXS8377 locus in the first group showed highest polymorphic allele in the Iraqi Arab population with the frequencies ranging from 0.005 to 0.16%. The power of discrimination (PD) value ranged from 0.663 for DXS7423 locus and 0.9066 for DXS8377 locus. In addition, the polymorphism information content (PIC) value ranged from 0.602974 for DXS7423 locus to 0.899206 for DXS8377 locus.

**Conclusions:**

Overall the X-STR markers become used as an important source of information beside the autosomal and Y-STR markers, especially for kinship testing and haplotype analysis.

## Background

The Forensic science is the collection of disciplines that scientifically contribute to the legal system; for instance, pathology, Odontology, anthropology, chemistry, toxicology, and genetics. Forensic genetics is the area in forensic science where DNA analysis is used for molecular identification of biological material found at crime scenes [1].

DNA analysis is now one of the most definitive techniques of identification in “forensic science”, paternity testing and missing individuals. It is a very effective technique of human identification [2, 3].

Short tandem repeats (STRs) are commonly utilized as DNA markers in paternity testing and criminal investigations due to their high genetic variation among individuals in population [4]. X-chromosome short tandem repeat (X-STR) markers are a significant addition to paternity and forensic casework. They've been beneficial in deficient paternity testing when the mother is accessible for typing. Both males and females retain one of their mother’s X chromosomes, and females retain their second X chromosome from their father. So, female individuals fathered by the same man share their paternal X chromosome and the other one X chromosome is the same with the mother [5]. Hence, in case of deficiency paternity in which the mother is available for typing, the possible X alleles of the putative father can be determined and the paternal profile can be reconstructed. Deficiency paternity cases, characterized by the absence of the alleged father, are a challenge for forensic genetics [6]. Furthermore, there are cases that show the effect of additional X-STR markers in identifying cases that cannot be solved using autosomal markers (e.g., special reverse paternity cases) [7].

There were 33 X-STR loci that have been used within the forensic community. As with autosomal STR loci used in forensic analysis, tetranucleotide repeats are most commonly selected due to lower stutter product formation compared to dinucleotide or trinucleotide repeats [8, 9].

The X-STR is a complementary tool to autosomal STR, (Y-STR) and (mtDNA) markers. It can be used in forensic investigations like complex kinship analysis [10]. DNA testing of X-chromosomal STR (X-STR) polymorphisms has been the main focus in a number of researches, primarily due to its applicability in the analysis of population genetic research by using multiplex polymerase chain reactions (PCR) for use in DNA testing in forensic application [11]. In other words, multiplex PCR is widely used for the study of population genetics, and as well as forensic [12, 13].

## Methods

### Study population

The population of this study includes 200 males apparently healthy unrelated participants from different region of Baghdad City, their ages ranged between 20 and 50 with mean age (36.83 ± 7.2) years. This study was conducted in a College of Biotechnology at Al-Nahrain University during the period from January 2019 to April 2020. Each participant was asked a systematic questionnaire for the various etiological factors of genetic disease, history of parents, relatives, and X-linked diseases such as hemophilia, G6PD dehydrogenase deficiency, and color blindness. This study was approved by the congress at the College of Biotechnology, Al-Nahrain University. Ethical consideration written consent was obtained from all participants and the researcher explained the objective of the study, signed written consent was taken from each individual participating in the study.

### Extraction of genetic material

Two milliliter venous blood samples were collected from participants into an EDTA tube for DNA extraction; sample was stored at − 23 °C until use. Total genomic DNA was extracted from frozen blood using the WIZPREP™ DNA Extraction Kit supplied by (Korea).

### Primers with fluorescent label

In this study 12 X-STR loci were achieved by using specific primers and fluorescent label listed in Table 1.

**Table 1:** Oligonucleotide primers and fluorescent label of loci used in the study

Target	Primer sequence fluorescent label (5′-3′)	Product size bp	Ta^**°**^C	Reference
DXS7424	F:5′AACACAGGAAGACCCCATC 3′ FAM R:3′GGCTAAGAAGAATCCCGCACA5′	76–100	58	[14]
HPRTB	F:5′TCTCTATTTCCATCTCTGTCTCC3′ FAM R:3′TCACCCCTGTCTATGGTCTCG 5′	144–176	58	
DXS8377	F: ′5CACTTCATGGCTTACCACAG3′ FAM R:3′GACCTTTGGAAAGCTAGTGT 5′	201–261	58	[15]
GATA31E08	F:5′CAGAGCTGGTGATGATAGATGA3′ VIC R:3′GCTCACTTTTATGTGTGTATG TATCTCC5′	101–129	58	[16]
DXS7423	F:5′GTCTTCCTGTCATCTCCCAAC 3′ VIC R:3′TAGCTTAGCGCCTGGCACAT A5′	179–191	58	
DXS8378	F:5′CACAGGAGGTTTGACCTGTT3′ VIC R:3′AACTGAGATGGTGCCACTGA 5′	198–222	58	[14]
DXS9895	F: 5′TTGGGTGGGGACACAGAG3′ NED R:3′CCTGGCTCAAGGAATTACAA 5′	139–163	58	[16]
DXS10074	F 5′ACTTCCTACTGCCCCACCTT3′ NED R:3′GTTTCCCCTCAGAGAGCTGA CACA5′	203–227	58	[17]
DXS6809	F:5'TGAACCTTCCTAGCTCAGGA3' NED R:3′TCTGGAGAATCCAATTTTGC5′	231–278	58	[16]
DXS7133	F5′AGCTTCCTTAGATGGCATTCA3′ PET R:3′GTTTTTAACGGTGTTCATGCT T5′	80–100	58	
DXS101	F:5′ACTCTAAATCAGTCCAAATATCT3′ PET R:3′AAATCACTCCATGGCACATG TAT5'	197–230	58	
DXS6807	F:5′GAGCAATGATCTCATTTGCA3′ PET R:3′AAGTAAACATGTATAGGAAA AAGCT5′	250–270	58	[18].

*Ta(C°)* annealing temp

### DNA allele size analysis

#### Amplification of DNA (PCR)

In this investigation, multiplex PCR was utilized to amplify the 12X-STR region (Table 2). Optimization of primer annealing temperature was performed by using gradient PCR, at different temperatures to identify the best conditions of primer annealing and using 0.5 Pmol concentrations for all primers [19]. The optimal temperature that gave the best results for PCR was 58 °C. The master mix components of PCR were prepared according to manufacturer’s recommendation in a GoTaq® PCR master mix of promega company. The PCR mixture was initially denatured at 95 °C for 12 min, and incubated for 30 cycles (denaturation for 1 min at 95 °C, annealing at 58 °C for 1 min and elongation for 3 min at 72 °C). The final elongation step was performed for 5 min at 72 °C.

**Table 2:** Locus information of X-STR markers

ChrX marker	Repeat motif	Allele range	PCR product
DXS 7424	TAA	7–20	76–100 bp
HPRTB	AGAT	6–19	144–176 bp
DXS8377	AGA	33–60	201–261 bp
GATA31E08	AGGG/AGAT	7–16	101–124 bp
DXS 7423	TCCA	8–19	179–195 bp
DXS 8378	CTAT	8–14	195–222 bp
DXS 9895	AGAT	11-19	102–160 bp
DXS 10074	AAGA	7–21	171–227 bp
DXS 6809	ATCT	27–40	231–278 bp
DXS 7133	ATAG	6–14	80–100 bp
DXS 101	CTT/ATT	14–32	197–230 bp
DXS 6807	GATA	11-17	250–270 bp

The PCR product was sent for to Macrogen Company (Korea), and then the PCR product size was compared with the information about X-STR reference allele and size. The microsatellite analysis was performed by Geneious Prime software.

### Power of discrimination (PD)

The probability that two randomly selected individuals will have different genotypes. Power of inclusion (Pi): sum of the squares of expected genotype frequencies. The following formula for calculating the power of discrimination in male according to [20].

### Polymorphism information content (PIC)

Refer to the value of a marker for detecting polymorphism within a population. The PIC using following formula according to [21]. It was used allelic frequencies marker.

### Statistical analysis

The Statistical Analysis System (SAS) (2012) program was used to detect the effect of different factors in study parameters.

## Results

### Amplification of X-STR

**Fig. 1:**
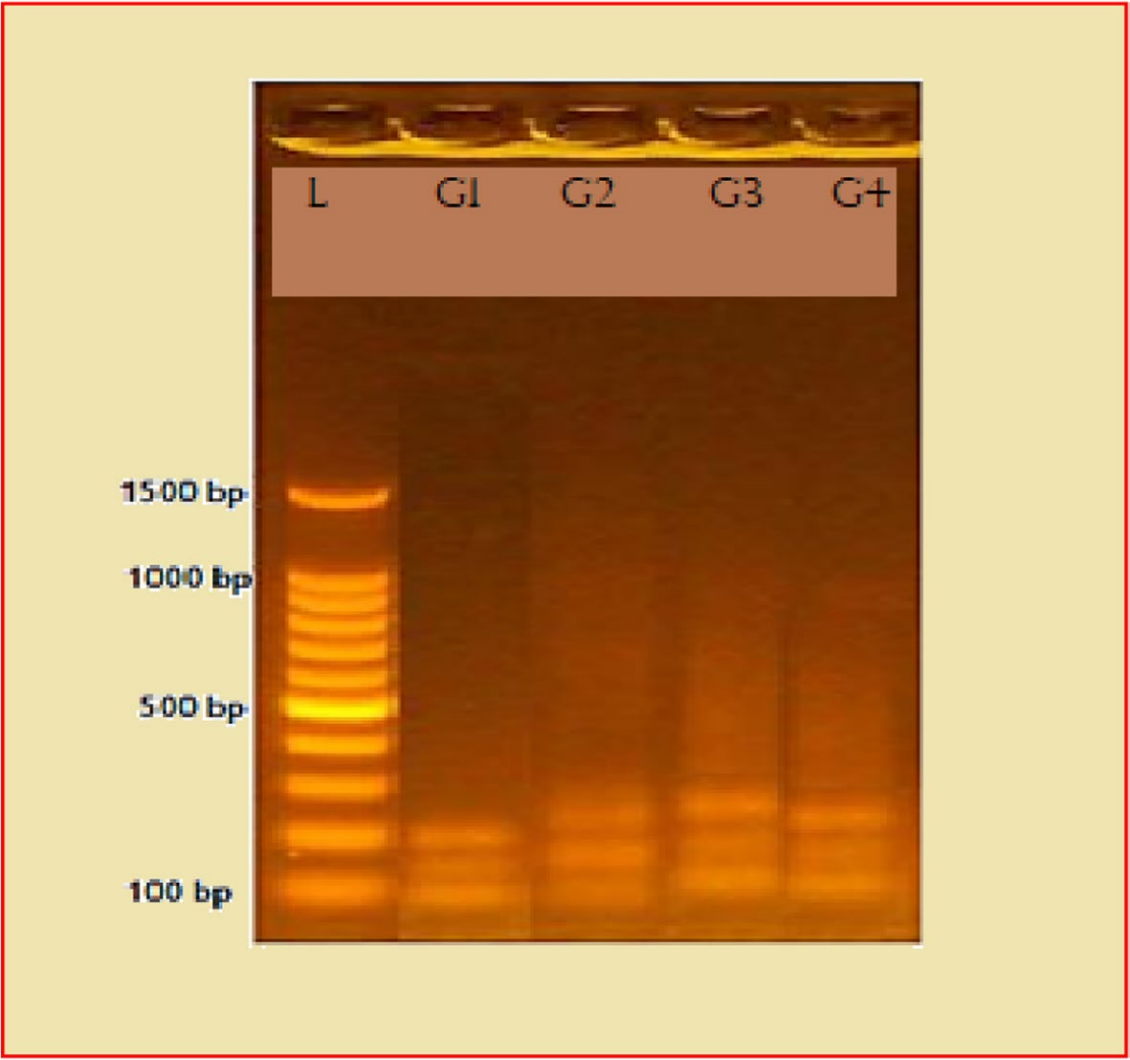
Agarose electrophoresis of multiplex PCR product using hot start Promega master mix. G1 represented first group (DXS7424, HPRTB, and DXS8377) gave PCR product size (100\150\200)bp; G2 represented second group (GATA31E08, DXS 7423, and DXS 8378) gave PCR product size (115\175\225)bp; G3 represented third group ( DXS9895, DXS10074, and DXS6809) gave PCR product size (125\185\250); G4 represented fourth group ( DXS7133, DXS101, and DXS6807) gave PCR product size (115\175\225)bp on 2.5% agarose at 70 V for 90 min (5 μl of PCR were loaded in each well).

After optimization of primers, the multiplex PCR was done for each three primers set at 58 °C, using hot start Promega master mix as shown in Fig. 1.

### Allele distribution and frequencies

#### The high, low, and rare allele

In the current study, twelve X-STR loci were grouped into four groups based on their molecular size (base pair). The first group includes DXS7424, HPRTB, and DXS8377. Second group involves GATA31E08, DXS7423, and DXS8378. The third group consists of DXS9895, DXS10074, and DXS6809. Fourth group includes DXS7133, DXS101, and DXS6807.

**Fig. 2:**
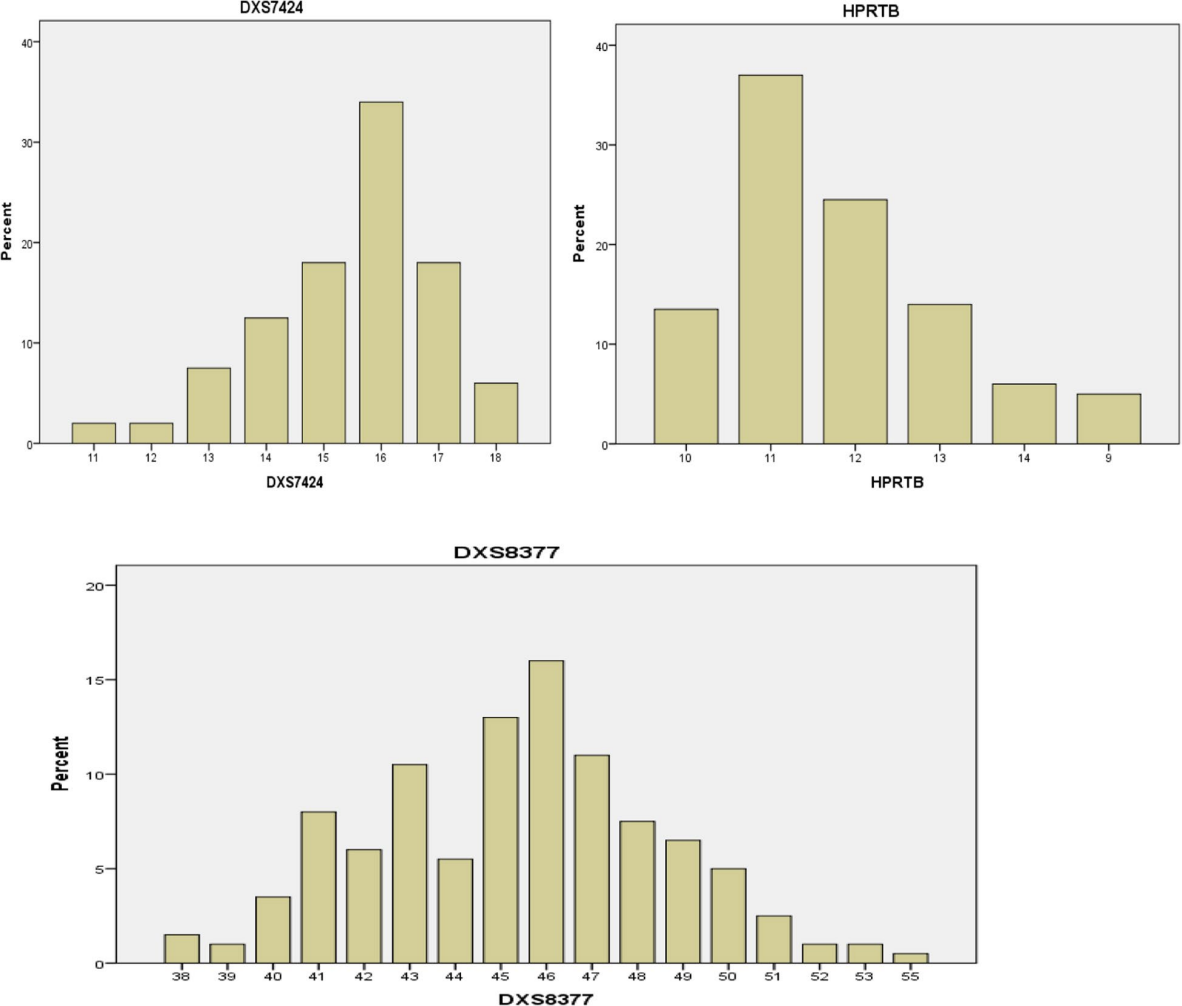
The percent distribution of allele frequency of the first group (DXS7424, HPRTB, and DXS8377) loci

**Fig. 3:**
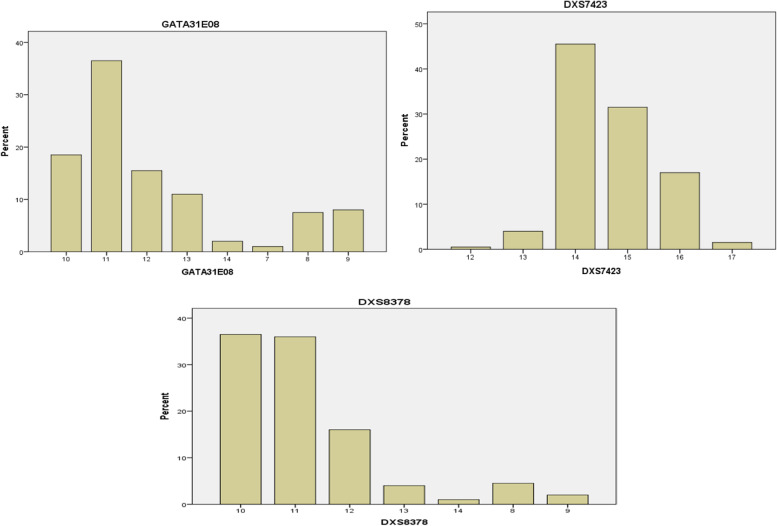
The percent distribution of allele frequency of second group (GATA31E08, DXS7423, and of DXS8378) loci

**Fig. 4:**
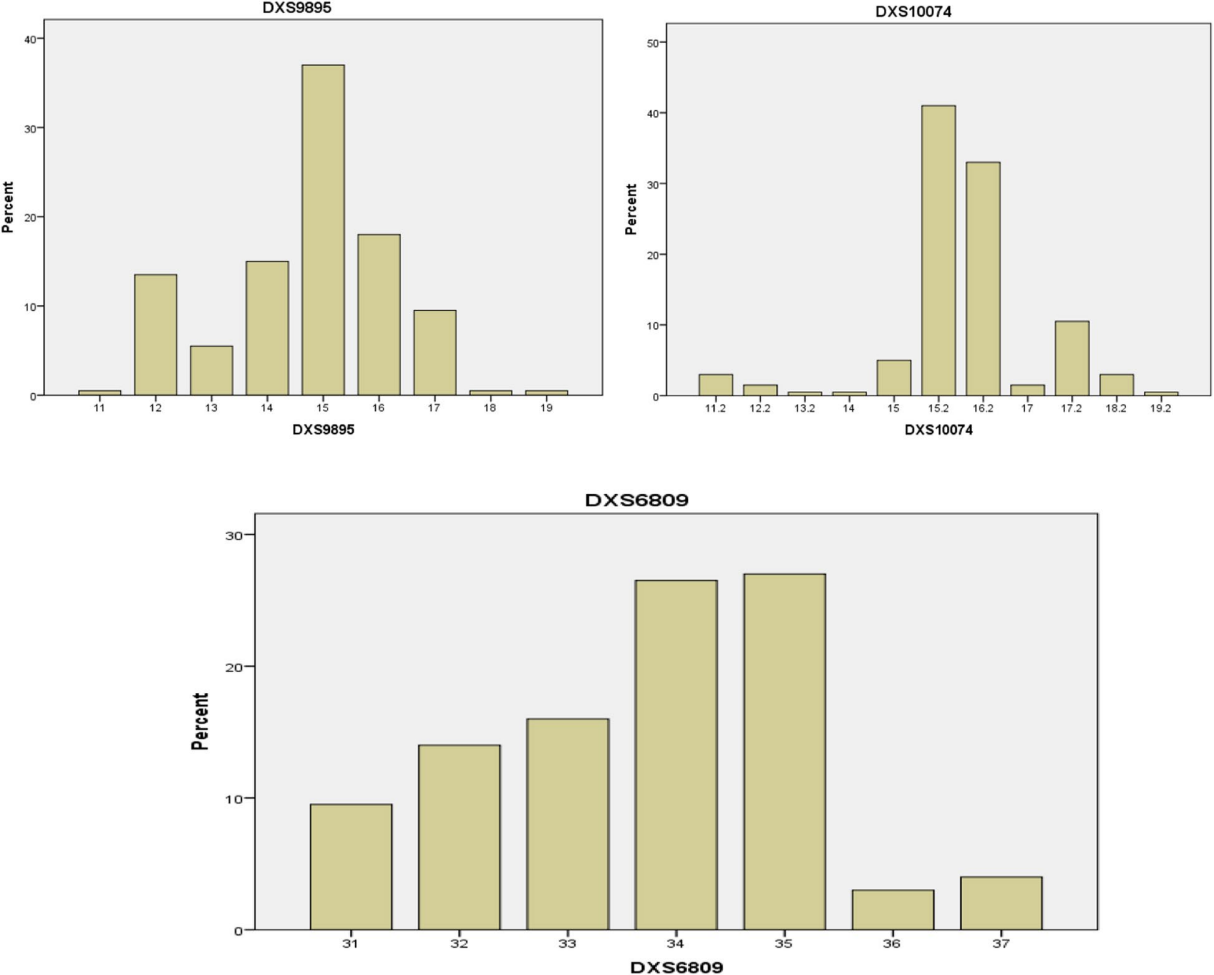
The percent distribution of allele frequency of the third group (DXS9895, DXS10074, and DXS6809) loci

**Fig. 5:**
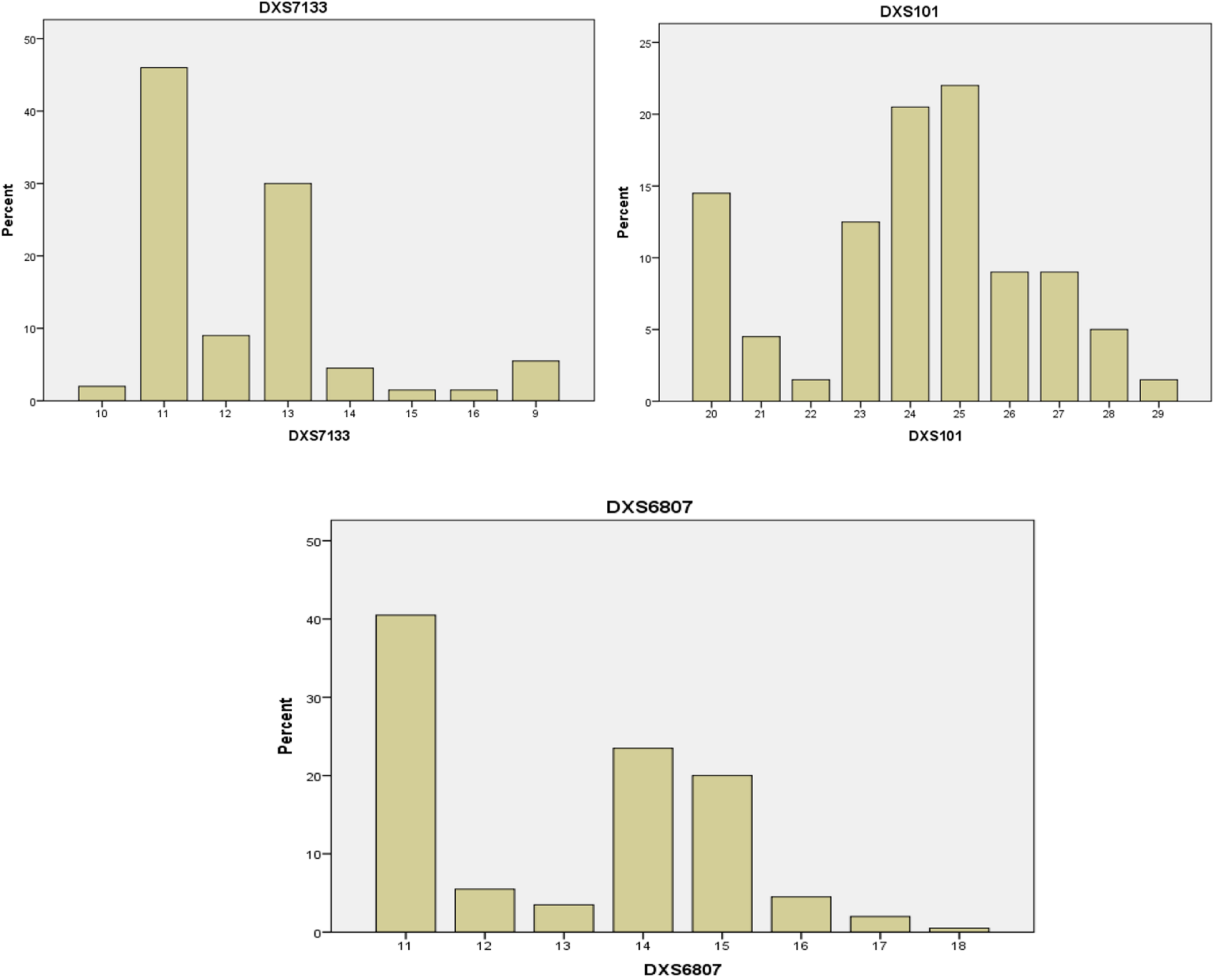
The percent distribution of allele frequency of the fourth group (DXS7133 DXS101 and DXS6807) loci

The distributions of the observed alleles and genotype frequencies for the 200 unrelated Arabic Iraqi males was shown in Table 3 and Figs. 2, 3, 4, and 5. The data from 200 unrelated Arabic Iraqi males was transformed to allele occurrence by counting the number of times of each allele that was identified and the results listed in Table 4.

**Table 3:** The percentage of allele frequencies of 200 unrelated Arabic Iraqi males from Baghdad Province

Allele	DXS7424%	HPRTB%	DXS8377%	GATA31E08 %	DXS7423%	DXS8378%	DXS9895%	DXS10074%	DXS6809%	DXS7133%	DXS101	DXS6807%
**7**				**0.01 L**								
**8**				**0.075**		**0.045**						
**9**		**0.05 L**		**0.08**		**0.02**				**0.055**		
**10**		**0.135**		**0.185**		**0.365 H**				**0.02**		
**11**	**0.02 L**	**0.37 H**		**0.365 H**		**0.36**	**0.005 R**			**0.46 H**		**0.405 H**
**11.2**								**0.03**				
**12**	**0.02 L**	**0.245**		**0.155**	**0.005 R**	**0.16**	**0.135**			**0.09**		**0.055**
**12.2**								**0.015 L**				
**13**	**0.075**	**0.14**		**0.11**	**0.04**	**0.04**	**0.055 L**			**0.3**		**0.035**
**13.2**								**0.005 R**				
**14**	**0.125**	**0.06**		**0.02**	**0.455 H**	**0.01 L**	**0.15**	**0.005 R**		**0.045**		**0.235**
**15**	**0.18**				**0.315**		**0.37 H**	**0.05**		**0.015 L**		**0.2**
**15.2**								**0.41 H**				
**16**	**0.34 H**				**0.17**		**0.18**			**0.015 L**		**0.045**
**16.2**								**0.33**				
**17**	**0.18**				**0.015 L**		**0.095**	**0.015 L**				**0.02 L**
**17.2**								**0.105**				
**18**	**0.06**						**0.005 R**					**0.005 R**
**18.2**								**0.03**				
**19**							**0.005 R**					
**19.2**								**0.005 R**				
**20**											**0.145**	
**21**											**0.045**	
**22**											**0.015 L**	
**23**											**0.125**	
**24**											**0.205**	
**25**											**0.22 H**	
**26**											**0.09**	
**27**											**0.09**	
**28**											**0.05**	
**29**											**0.015 L**	
**30**												
**31**									**0.095**			
**32**									**0.14**			
**33**									**0.16**			
**34**									**0.265**			
**35**									**0.27 H**			
**36**									**0.03 L**			
**37**									**0.04**			
**38**			**0.015**									
**39**			**0.01 L**									
**40**			**0.035**									
**41**			**0.08**									
**42**			**0.06**									
**43**			**0.105**									
**44**			**0.055**									
**45**			**0.13**									
**46**			**0.16 H**									
**47**			**0.11**									
**48**			**0.075**									
**49**			**0.065**									
**50**			**0.05**									
**51**			**0.025**									
**52**			**0.01 L**									
**53**			**0.01 L**									
**55**			**0.005 R**									

*H = high allele frequency, *L* = low allele frequency, *R* = rare allele frequency

**Table 4:** The allele occurrence for the 200 unrelated Arabic Iraqi male population

Allele	DXS7424	HPRTB	DXS8377	GATA31E08	DXS7423	DXS8378	DXS9895	DXS10074	DXS6809	DXS7133	DXS101	DXS6807
**7**				**2 L**								
**8**				**15**		**9**						
**9**		**10 L**		**16**		**4**				**11**		
**10**		**27**		**37**		**73 H**				**4**		
**11**	**4 L**	**74 H**		**73 H**		**72**	**1 R**			**92 H**		**81 H**
**11.2**								**6**				
**12**	**4 L**	**49**		**31**	**1 R**	**32**	**27**			**18**		**11**
**12.2**								**3 L**				
**13**	**15**	**28**		**22**	**8**	**8**	**11 L**			**60**		**7**
**13.2**								**1 R**				
**14**	**25**	**12**		**4**	**91 H**	**2 L**	**30**	**1 R**		**9**		**47**
**15**	**36**				**63**		**74 H**	**10**		**3 L**		**40**
**15.2**								**82 H**				
**16**	**68 H**				**34**		**36**			**3 L**		**9**
**16.2**								**66**				
**17**	**36**				**3 L**		**19**	**3**				**4 L**
**17.2**								**21**				
**18**	**12**						**1 R**					**1 R**
**18.2**								**6**				
**19**							**1 R**					
**19.2**								**1**				
**20**											**29**	
**21**											**9**	
**22**											**3 L**	
**23**											**25**	
**24**											**41**	
**25**											**44 H**	
**26**											**18**	
**27**											**18**	
**28**											**10**	
**29**											**3 L**	
**30**												
**31**									**19**			
**32**									**28**			
**33**									**32**			
**34**									**53**			
**35**									**54 H**			
**36**									**6 L**			
**37**									**8**			
**38**			**3**									
**39**			**2 L**									
**40**			**7**									
**41**			**16**									
**42**			**12**									
**43**			**21**									
**44**			**11**									
**45**			**26**									
**46**			**32 H**									
**47**			**22**									
**48**			**15**									
**49**			**13**									
**50**			**10**									
**51**			**5**									
**52**			**2 L**									
**53**			**2 L**									
**55**			**1 R**									

*H = high allele frequency, *L* = low allele frequency, *R* = rare allele frequency

##### First group

The alleles found at DXS7424 locus were 11, 12, 13, 14, 15, 16, 17, and 18 with allele frequency range from 0.02 to 0.34 and the allele 16 appeared as high-frequency allele while the low-frequency alleles were allele 11and 12.

The alleles found at HPRTB locus found were 9, 10, 11, 12, 13, and 14 with allele frequency range from 0.05 to 0.37 for allele 11 appeared as high frequency and the allele 9 found as low-frequency allele.

Alleles found at the DXS8377 locus were 38, 39, 40, 41, 42, 43, 45, 46, 47, 48, 49, 50, 51, 52, 53, and 55 with the allele frequency ranging from 0.005 to 0.16. Allele 46 was the high-frequency allele while the low allele frequency were alleles 39, 52, and 53 and the rare allele was allele 55.

##### Second group

The alleles found at GATA31E08 locus were 7, 8, 9, 10, 11, 12, 13, and 14 with allele frequency ranging from 0.01 to 0.365 and the allele 11 was found to be a very common and the low frequency was found in allele 7.

The alleles detected at the DXS7423 locus were 12, 13, 14, 15, 16, and 17 with allele frequency ranging from 0.005 to 0.455, and allele 14 appearing as high frequency and allele 17 in low occurrences while allele 12 found as rare allele.

The alleles showed at DXS8378 locus were 8, 9, 10, 11, 12, 13, and 14 that have allele frequency range from 0.01 to 0.365 and the allele 10 appeared in a high frequency and allele 14 was observed in low frequency.

##### Third group

The alleles that detected at DXS9895 locus were 11, 12, 13, 14, 15, 16, 17, 18, and 19 with a allele frequency range of 0.005–0.37 and the allele 15 was found as a high frequency, and low frequency was found in allele 13, whereas the alleles 11, 18, and 19 were revealed as a rare alleles.

The alleles that showed at DXS10074 locus were 11.2, 12.2, 13.2, 14, 15, 15.2, 16.2, 17, 17.2, 18.2, and 19.2 with their allele frequency ranged from 0.005 to 0.41 and the allele 15.2 was found to be the higher one while the alleles 12.2 and 17 were found in a low frequency but the alleles 13.2, 14, and 19.2 were observed as a rare alleles.

The alleles found at DXS6809 locus were 31, 32, 33, 34, 35, 36, and 37 with their percentage frequency range from 0.03 to 0.27 and the allele 35 was found the higher in occurrence and allele 36 found as the lowest allele.

##### Fourth group

The DXS7133 locus was found with their alleles 9, 10, 11, 12, 13, 14, 15, and 16; their frequency from 0.015 to 0.46 and the allele 11 was found in a higher repeats while allele 15 and 16 found in low repeats. The alleles detected at DXS101 locus were 20, 21, 22, 23, 24, 25, 26, 27, 28, and 29 with a percentage of the frequency range from 0.015 to 0.22 and the allele 25 was found in high frequency. The allele repeats detected in the DXS6807 locus were 11, 12, 13, 14, 15, 16, 17, 18) with frequencies ranging from 0.005 to 0.405, and allele 11 appearing typically and allele 17 found in a low allele frequency but allele 18 consider as rare allele.

### The power of discrimination (PD)

The PD value ranged from 0.663 for DXS7423 locus and 0.9066 for DXS8377 locus as results shown in the Table 5.

**Table 5:** Forensic efficiency parameter: power of discrimination (PD) of 12 XSTR loci for 200 Arab Iraqi populations

Locus	Power of discrimination
**DXS7424**	**0.79395**
**HPRTB**	**0.75915**
**DXS8377**	0.9066*
**GATA31E08**	**0.7839**
**DXS7423**	0.663*
**DXS8378**	**0.70745**
**DXS9895**	**0.77785**
**DXS10074**	**0.70715**
**DXS6809**	**0.80015**
**DXS7133**	**0.6844**
**DXS101**	**0.85175**
**DXS6807**	**0.73405**

*The significance value of power of discrimination (> 0.05)

### Polymorphism information content (PIC)

The value ranged from 0.602974 for DXS7423 locus to 0.899206 for DXS8377 locus, the results listed in the Table 6.

**Table 6:** Forensic efficiency parameter polymorphism information content (PIC) of 12 X-STR loci for 200 Arab Iraqi populations

Locus	Polymorphism information content
DXS7424	0.767245
HPRTB	0.724221
DXS8377	0.899206*
GATA31E08	0.756917
DXS7423	0.602974*
DXS8378	0.657072
DXS9895	0.74922
DXS10074	0.661635
DXS6809	0.77158
DXS7133	0.63775
DXS101	0.834708
DXS6807	0.69489

*The significance value of polymorphism information content (> 0.05)

## Discussion

Over the last decade, the usage of X chromosomal short tandem repeat (STR) markers has increased dramatically in the forensic field. This current study was conducted to analyze the genetic of (X-STR) frequencies in Arab Iraqi male population. The purpose of this study was to explore at the frequency of 12 (X-STR) haplotypes in the Iraqi population as a reference data source for individual identification. As allele frequency, the distributions of observed alleles and genotype frequencies for the 200 unrelated Arab Iraqi males were demonstrated. The results of the present study revealed the high and low frequency of these alleles. Our findings revealed that the DXS7424 locus of Iraqi males showed the range between 0.02 and 0.34 with high frequency for allele 16 and low frequency for alleles 11 and 12. Relative to the DXS7424 locus, previous study by Nakamura Y and Minaguchi K., 2010 showed that the alleles in Japanese population were 12, 13, 14, 15, 16, 17, and 18 and frequency ranged from 0.011 to 0.445 with similar high frequency (0.445) for allele 16 [16].

According to the above results concerning HPRTB locus, the allele frequency ranged from 0.05 to 0.37 with high and low frequency for allele 11 and allele 9 respectively. The study by Hameed et al. 2015 conducted in Iraq found that the alleles in Iraqi population were 7, 8, 9, 10, 11, 12, 13, 14, 15, and 16 and frequency ranged from 0.002 to 0.436 with high frequency (0.436) for allele 13 [22]. This finding could be related to the use of argus 8X-STR specific X-STR kits, whereas in this study, the authors employed multiplex PCR from 12 loci as described by Nakamura Y and Minaguchi K ., 2010 [16].

In the DXS8377 locus, the high, low, and rare frequency alleles were for allele 46, alleles 52, 53, and allele 55 respectively with frequencies ranged from 0.05 to 0.16. However, the findings by Poetsch et al. 2005 discovered that the alleles at this locus were 39, 40, 41, 42, 43, 45, 46, 47, 48, 49, 50, 51, 52, 53, 54, 55, and 56 in German population and the allele 51 had a high-frequency percentage (0.138) [14].

Regarding to the GATA31E08 locus, the high and low allele frequency alleles were observed in allele 11 and 7 respectively and the allele frequency ranged from 0.01 to 0.365. It was found by Nakamura Y and Minaguchi K., 2010 that the most prevalent alleles in the Japanese population were 7, 8, 9, 10, 11, 12, 13, and 14, with a frequency ranging from 0.001 to 0.307, with allele 11 having the highest frequency (0.307) [16].

In the present study, the DXS7423 locus had a high and low allele frequency showed in allele 14 and 17 with a rare allele showed in allele 12. Similarly, previous studies done by Al-Snan et al. 2019 and Hameed et al. 2015 reported that the allele 14 at DXS7423 locus emerged as a high-frequency allele with allele frequency 0.462 and 0.409, and this locus exhibit low polymorphic alleles [22, 23]. The study done by Nakamura Y and Minaguchi K., 2010 showed that the alleles in Japanese population was 13,14,15,16 with frequency ranged from 0.005 to 0.608 with high frequency (0.608) for allele 15 [16].

As a result, the DXS8378 locus had a high and low allele frequency for allele 10 and allele 14 with a range from 0.01 to 0.365. In a contrast with the present study, previous studies in Iraq and Germany revealed that the allele 11 having the highest frequency (0.371 and 0.374) respectively [22].

The DXS9895 locus showed the high allele frequency for allele 15 and low frequency for allele 13 with a rare allele reported for allele 11, 18, and 19. Such observation suggested by Poetsch et al. 2005 upon the DXS9895 locus found that the allele 24 having a high frequency [14].

The allele frequencies at DXS10074 locus ranged from 0.005 to 0.41 and the allele 15.2 appeared in high frequency and allele 12.2 and 17 in low frequency and as a rare allele found in allele 13.2, 14, and 19.2. A previous study done in Iraqi and Turkish population revealed that the high frequency allele at the DXS10074 locus was 15 [22, 24].

The allele frequencies at DXS6809 locus ranged from 0.03 to 0.27, and the allele 35 appeared in a high frequency while the low frequency appeared in allele 36. Such a result of previous study in Japan revealed that the allele repeats in Japanese population was 26, 29, 30, 31, 32, 33, 34, 35, 36, and 37 and frequency range from 0.003 to 0.309 with a high frequency for allele 33 [16].

The allele frequencies at DXS7133 locus ranged from 0.02 to 0.46 and the allele 11 appeared in a high frequency while the low frequency was found in alleles 15 and 16. The findings obtained by Nakamura Y and Minaguchi K., 2010 conducted in japan showed that the allele repeat in Japanese population was 9, 10, 11, and 12 and frequency range from 0.06 to 0.7 with a high frequency for allele 9 [16].

The allele frequencies at DXS101 locus ranged from 0.015 to 0.22 and the allele 25 appeared in high frequency with low frequency observed in allele 22 and 29. The result of previous study in Germany showed that the allele repeat in German population was 14, 15, 16, 17, 18, 19, 20, 21, 22, 23, 24, 25, 26, 27, 28, 29, and 30 and frequency range from 0.03 to 0.2 with high frequency for allele 24 [14].

The allele frequencies at DXS6807 locus ranged from 0.005 to 0.405 and the allele 11 appeared in high frequency while the low allele frequency showed in allele 17 and allele 18 found as a rare allele. A prior investigation carried out in japan showed that the allele repeat in Japanese population was 11, 12, 13, 14, and 15 and frequency range from 0.08 to 0.370 with similar a high frequency for allele 11 [16].

Rare alleles are polymorphic alleles that occur in less than 1% of the population. However, the frequency of detecting functional uncommon alleles was highly dependent on the sample size [25].

The power of discrimination (PD) is the chance that choosing two individuals randomly would not have matching DNA profile. The current study found the PD value ranged from 0.663 for DXS7423 locus and 0.9066 for DXS8377 locus. This result is close to Iraqi study that showed the DXS7423 locus had 0.522 of PD value [22] and an Italian study the PD value was 0.913 for DXS8377 locus [26].

Polymorphism information content (PIC) is evaluated by the markers ability to identify polymorphism in the population based on the number of alleles detected and the frequency of their distribution. Therefore, PIC determines the marker’s discriminatory capability, basically depending on the number of recognized (identified) alleles and their frequency of distribution [21]. The result of the present investigation revealed that the PIC for all twelve loci were appeared more than 0.5, indicating good informativeness of all X-STR markers.

In order for novel alleles to be noted in DNA profiles, consideration should be given to these new alleles for forensic science recommendations with a view to being included in the DNA database, and in order to assign frequency to estimate the probability of a specific DNA profile across a population of interest (random match probability) [27, 28].

The distribution occurrence is another measure to examine the frequencies of the most common occurrence genotypes in order to identify the usefulness for particular set of DNA markers would therefore be the least powerful in terms of being able to differentiate between two unrelated individuals [29, 30].

The X-STR loci become main completely and alternative uses for forensic application especially, kinship testing, miss distress, and more efficient use degraded DNA and is widely used in many complicated cases.

## Conclusions

Our result found that in Iraq Arab population, the highest forensic efficiency parameter was DXS8377 locus that has the highest polymorphic allele. In contrast, the DXS7423, DXS9895, DXS10074, and DXS6807 loci have a rare allele with such have a little role in the Iraqi population database. The finding of the present study is relevant to major concerns in forensic science, such as population geneticists’ inquiry into the behavior of rare variants, with an emphasis on alleles with low relative frequency. As a recommendation in this study, we suggested to focus the level of X-STR loci polymorphism in a three major ethnic groups in the Iraq to start for building X-STR data base.

## Data Availability

All data generated or analyzed during this study are included in this published article.

## Abbreviations

STR: Short tandem repeat
X-STR: X-chromosome short tandem repeat
PCR: Polymerase chain reaction
mtDNA: Mitochondrial DNA
Y-STR: Y-chromosome short tandem repeat
EDTA: Ethylenediaminetetraacetic acid
Pi: Power of inclusion
PIC: Polymorphism information content
ISFH: International Society for Forensic Haemogenetics
PD: The power of discrimination
